# Patient Perceptions of Dermatologic Photography: Scoping Review

**DOI:** 10.2196/33361

**Published:** 2022-01-26

**Authors:** William Kim, Torunn Sivesind

**Affiliations:** 1 Department of Dermatology University of Colorado School of Medicine Aurora, CO United States

**Keywords:** patient perceptions, patient perspectives, medical photography, clinical photography, dermatology, skin disease, dermatologic photography, medical images, skin of color, SOC

## Abstract

**Background:**

Medical photography is used extensively in dermatology to record disease progression, measure treatment response, and help teach patients about skin disease; such photos are also commonly utilized in teledermatology, medical education, research, and medical reference websites. Understanding patient perceptions of medical photographs obtained during dermatologic care in the clinic or hospital setting is critical to enable the delivery of high-quality, patient-centered medical care.

**Objective:**

The aims of this study were to elucidate patient perceptions of skin photos in dermatology and to explore possible next steps in improving the patient experience with medical photography in the hospital or clinic setting.

**Methods:**

A scoping review of the literature was performed using the PubMed database, with clinic- or hospital-based full-text publications in English spanning the last 10 years considered for inclusion.

**Results:**

The majority of included studies (10/11, 91%) found positive patient attitudes toward medical photographs. The majority of patients (1197/1511, 79.2%) felt that medical photographs could improve medical care in the clinic setting. Written consent detailing all photo uses, including secondary uses (such as research or teaching), was preferred, apart from in 1 study. Patients preferred or found it acceptable for the photographer of their medical photos to be a physician (1301/1444, 90.1%). Clinic-owned cameras with departmental record storage were the preferred modality. Latinx and African American patients expressed less trust in the utility of medical photographs to improve care, compared with Asian and White patients. The minimal number of available publications on this topic and the inclusion of articles older than 5 years are limitations, since patient perceptions of medical photography may have rapidly changed during this time span, particularly in light of the COVID-19 pandemic and the subsequent increase in teledermatology visits.

**Conclusions:**

Patients reported positive perceptions of dermatologic photography for improving their medical care. Ethnic disparities in patient perceptions require further exploration to better elucidate nuances and develop interventions to improve the experience of marginalized patients. Building patient trust in nonphysician photographers may enhance clinic efficiency. Although clinic-owned cameras are well-accepted by patients, improved patient education surrounding the safety of electronic medical record phone applications is needed.

## Introduction

Dermatology is a medical field that prioritizes visualization of pathology. One important tool to aid this visualization is medical photography. Medical photographs can be used to record disease progression, measure response to treatments, and help teach patients about skin disease [1-3]. In addition to their use in the clinical setting, medical photographs are used to teach medical students, dermatologists, and other health care workers about skin disease. Medical photographs may also be incorporated into research and medical reference websites [4].

Despite its prevalence in dermatology, few studies have examined how patients feel about medical photography in the hospital or clinic setting and whether discrepancies exist in patient perceptions of medical photography among various people, based on factors such as ethnicity, socioeconomic status, gender, and sexual orientation. Given the significant utilization of medical photography in daily dermatology practice, understanding patients’ perceptions of this tool is necessary to achieve high-quality, patient-centered care. We therefore performed a scoping review of the literature to assess patient perceptions of skin photos in dermatology and to explore possible next steps in improving the patient experience with medical photography in the hospital or clinic setting.

## Methods

A literature review was performed using the PubMed database. The following search string was utilized: (“Dermatology”[Mesh] OR “Skin Diseases”[Mesh] OR “skin*” OR “derm*”) AND (“photography*” OR “picture*”) AND (“perception*” OR “attitude*” OR “perspective*” OR “feel*” OR “satisfaction*” OR “acceptance*”) AND (“patien*” OR “provider*” OR “clinician*”). All available full-text publications in English spanning the last 10 years involving patient perceptions of medical photography in a dermatology clinic or hospital setting were included. Studies largely focused on patient perceptions of teledermatology or in nondermatology settings were excluded from our study tables.

## Results

We identified and selected 11 studies for inclusion after screening the abstracts of 468 articles. Table 1 includes a summary of the 11 articles and their primary findings surrounding patient perceptions of medical photography in dermatology. Table S1 in Multimedia Appendix 1 provides further granularity, categorizing perceptions by category: consent, photographer role and badge, gender, photograph capture method, image storage, image use and identifiers, mental well-being and trust, and ethnic variations.

**Table 1:** Summary of included publications (2011-2021) with principal findings.

Article title	Author(s)	Year	Study location	Study setting	Study sample size	Perceptions of medical photography in dermatology
Patients’ acceptance of medical photography in a French adult and paediatric dermatology department: a questionnaire survey	Hacard et al [5]	2013	France	Inpatient hospital	N=272 (158 adults and 114 children)	Positive perceptions by adult patients (99.3%) and parents of pediatric patients (96.0%)
Patient perspectives on medical photography in dermatology	Leger et al [4]	2014	New York, NY	Inpatient hospital (2), outpatient clinic (2)	N=398	Positive perceptions (88.7%) for patient medical care
Patient perception on the usage of smartphones for medical photography and for reference in dermatology	Hsieh et al [1]	2015	Chicago, IL	Inpatient hospital, outpatient clinic	N=300	Positive perceptions when used for patient care: charting (84.8%) and treatment/disease monitoring (82.1%)
Total body photography as an aid to skin self-examination: a patient’s perspective	Secker et al [6]	2016	Leiden, Netherlands	University hospital	N=179	Neutral perceptions for total body photos as being useful (44.7%)
Smartphones in the dermatology department: acceptable to patients?	Soriano et al [7]	2017	London, England	Hospital (3)	N=203	Positive perceptions for medical photography of skin lesions by patient smartphone or hospital camera
Perception and acceptability of medical photography in Chinese dermatologic patients: a questionnaire survey	Wang et al [8]	2017	China	Outpatient clinic	N=474	Positive perceptions (79.9%) for improving care (diagnosis and treatment)
Attitudes to medical photography: study of a Spanish population at the Pius Hospital de Valls in Tarragona, Spain	Pasquali et al [9]	2019	Tarragona, Spain	Outpatient setting	N=134 (100 dermatology patients)	Positive perceptions for medical uses (94.8%)
Smartphones in dermatology: acceptance of smartphone photography by the informed patient	Accetta et al [10]	2020	Buffalo, NY; New Orleans, LA	Outpatient setting	N=400 (200 from each location)	Positive perceptions (95.5%) of medical photography
Patients’ experiences and attitudes of using a secure mobile phone app for medical photography: qualitative survey study	Wyatt et al [11]	2020	Rochester, MN	18 departments including dermatology	N=71 (19 dermatology patients)	Positive perceptions of a secure EHR^a^-integrated (PhotoExam) application for medical care (67%) and would recommend to others (74%)
Study of patients’ satisfaction toward photographing their skin lesions for educational purposes	Amirian et al [12]	2021	South Iran	Hospital	N=200	Positive perceptions, with majority (67.5%) satisfied with medical photography of skin lesions
Evaluation of standardized scalp photography on patient perception of hair loss severity, anxiety, and treatment	Pathoulas et al [3]	2021	Boston, MA	Outpatient setting	N=119	Positive perceptions of scalp photography as being helpful (98.3%) and increasing motivation (98.3%) to complete alopecia treatment

^a^EHR: electronic health record.

## Discussion

### Principal Findings

Overall, the majority of included studies (10/11, 91%) found positive patient attitudes toward medical photographs [1,3-5,7-12]. Additionally, many dermatology patients (1197/1511, 79.2%) felt that medical photographs could improve their care, diagnosis, or treatment in the clinical setting [3-6,8,11]. These positive patient attitudes of studies from diverse locations (including the United States, France, Spain, South Iran, China, and the United Kingdom) are reassuring that medical photographs are generally well-accepted by dermatology patients. Patient perceptions of several key aspects of medical photography in dermatology are discussed in further detail in the following sections.

### Consent

The most recent US-based study (2020) [11] reported a slight patient preference for verbal over written consent, although prior studies indicated a preference for written consent [1,4,11]. Patients in China and France (adult population) had nearly equivalent preferences for oral or written consent [5,8]. Differences in cultural norms, survey question wording, and study population may have influenced these results. Given these geographical variations in consent preference and the possibility of intraregional variations, it is beneficial to obtain both oral and verbal photo consent, when feasible. 

A standardized dermatologic medical photography consent form written in plain language that incorporates current research should be developed, detailing all possible medical photograph uses. An accompanying form for providers should also be developed and provide tips to improve the patient experience, along with ethnic disparities of which to be mindful [4,5,8,12]. A tiered consent form is currently being studied, “allowing patients to consent for use of photographs for (1) clinical care only; (2) clinical care and internal education; or (3) clinical care, internal education, and external education” [11]. An educational photograph booklet may also help improve patient satisfaction with medical photography but requires further research [13].

### Photographer Role, Gender, and Identification

In general, patients seem to prefer physicians to act in the role of photographer (1301/1444, 90.1%)—apart from the findings of 3 studies that reported more equitable or indifferent opinions regarding who should assume the photographer role (physician, hospital staff, or professional photographer) [4,5,7-9,11,12]. Patient preferences for photographer gender varied based on study location [4,8]. Male patients provided greater consent for photo uses [12]. These preference variations regarding photographer role and gender may be influenced by societal perceptions of health care workers and gender-related patient experiences.

Leger et al [4] pointed out the necessity of strengthening overall patient trust in “nonphysician photographers and in physicians of the opposite gender.” Improving patient trust in photographers of the opposite gender and in nonphysician photographers can enhance patient comfort, patient compliance, and clinic efficiency [4]. Part of ensuring patient trust in the medical photography process is having the photographers wear identifiable badges so patients know the clinical role of the photographer [5,8].

### Image Capture and Storage

Most patients favored a clinic- or hospital-owned camera or patient personal phone rather than a physician’s personal camera or cell phone for medical photographs, although one study reported findings of patient indifference with a mobile device versus a professional camera [1,4,5,7,8,11,12]. Patient concerns with the use of mobile phones were related to confidentiality, poor professionalism, and automatic photo uploading [1,5]. However, patients found smartphones acceptable to reference information when providing patient teaching, and 1 study reported a 79% acceptance rate for smartphones for medical photography after an information sheet detailing secure storage was provided [1,10]. Given the presence of electronic medical record (EMR) applications designed for cell or mobile phone use—with protection measures in place—it may be worthwhile to explain the security of using one’s cell phone with an EMR application for photo capture, possibly with an information sheet, as this is highly conducive to efficiency and confidentiality [11]. 

Patients preferred and were satisfied with image storage within departmental records [5,7,8]. One storage solution for maximal confidentiality and protection is an EMR cloud-based storage system, such that photos are not stored locally on a physician’s personal computer or phone [4]. An example is a mobile phone point-of-care application that safely uploads a medical photo to the patient’s chart without saving the photograph to the physician’s phone; 67% of patients felt this application improved patient care [11]. Alternatively, a clinic-owned camera that is used to take all patient photos, stays in the exam room, and is uploaded daily to patient charts is another reasonable option. 

### Image Uses and Identifiers 

Patients were more comfortable with their photographs being used for diagnosis and treatment (including teledermatology), teaching, and research purposes [1,4,5,8,11]. One study reported patient attitudes towards scalp photography as useful, increasing motivation for treatment and improving alopecia-associated anxiety [3]. Patients were more comfortable and willing to allow secondary image use such as educational purposes when photos were unidentifiable [4,9,11]. For image uses external to the clinical setting, patients felt more comfortable with scientific publications or case discussion than with health websites [5,8]. Public health campaigns to strengthen patient trust in the use of medical photography for dermatologic websites (such as VisualDx, Dermnet, and even Wikipedia) can be beneficial. Greater incorporation of high-quality patient photographs into these web-based reference sites has the potential to improve education for both providers and patients. Ensuring the inclusion of dermatologic photos of all Fitzpatrick skin types is necessary to eliminate existing disparities related to skin of color (SOC) and to promote more equitable representation on these websites [14]. 

### Body Region 

The majority (348/398, 84.7%) of patients felt comfortable with their deidentified photos being used for teaching, and this rate decreased when involving an intimate body area (232/398, 58.3%) [4,9]. In general, patients were less comfortable with medical photography of genital regions [7]. A possible solution to improve patient comfort when involving an intimate body area includes an easily understandable, standardized consent form listing all possible image uses and verbally explaining that these images will be confidential. 

### Mental Well-being and Trust

Among the included studies, there were more missing data responses for negative perception questions, and about 5% of patients felt discomfort with medical photography [5,8]. Patients may feel intimidated to say “no” to a physician out of concern for subtle retaliation in care; ensuring that patients have the autonomy and space to say “no” to medical photography can foster a safe environment for patients and strengthen the patient-physician relationship.

Medical photography may be utilized to track patient response to treatments and has been shown to reduce disease-associated anxiety, although some patients reported feeling shame around photos [3,6]. Allowing patients to see their own medical photographs may contribute to better patient outcomes by strengthening trust, improving the patient-physician relationship, and increasing patient education and treatment satisfaction [3,5,8].

### Ethnic and Age Variations

Latinx, African American, and Afro-Caribbean patients were more likely to believe medical photography would fail to improve their care and expressed greater discomfort with medical photography [4,7]. Among a multitude of related findings (Table S1 in Multimedia Appendix 1) was the discovery that White patients reported the least discomfort with medical photography [4].

Negative health care experiences by Latinx and African American patients may be due to systemic inequities and implicit biases in health care [4]. One study reported that Hispanic and Black patients were significantly less likely to receive medical outpatient care for a dermatologic disease [15]. If the process of medical photography contributes to distress for these patients, they may be less likely to seek dermatologic care, contributing to later diagnosis and more advanced skin cancers at time of first presentation for African American and Hispanic patients [16]. Thus, African American and Latinx patient perceptions of medical photography are of critical importance in promoting health equity.

Ethnic differences in perceptions should also be addressed to improve representation of SOC patients in dermatologic photography. Existing studies have categorized ethnic groups into broad categories such as African American, Latinx, Asian, and White, but further studies, (possibly including quality improvement studies) need to be done with more categorized ethnic groups such as Mexican, Puerto Rican, Chinese, Vietnamese, and others in order to better understand patient perceptions of medical photography in dermatology for various ethnic groups [4,7].

Skin diseases can appear visually different in SOC individuals compared with non-SOC individuals [17]. If patients with darker skin are uncomfortable having dermatologic photos taken, it limits the available number of photos of darker skin tones, hindering dermatologic education by not exhibiting the entire scope of skin disease presentations and contributing to incorrect diagnoses.

The relative lack of ethnic diversity among dermatology providers is another barrier—one solution to improve non-White patient comfort and trust in medical photography is to increase provider ethnic diversity within dermatology [18]. Future research on improving SOC patient perceptions of medical photography will improve the number and quality of SOC photographs, thus bolstering the accessibility and applicability of information related to skin disease presentations and improving health outcomes for non-White patients.

### Teledermatology and COVID-19

The COVID-19 pandemic has drastically shifted teledermatology rates: 96.9% of dermatologists utilized teledermatology during the pandemic compared with 14.1% prior to COVID-19 [19]. Preliminary patient perceptions of teledermatology (using a patient’s webcam or mobile phone to document skin disease presentation, progression, and treatment response) indicate patient satisfaction with teledermatology despite a preference for in-person visits; further exploration of this topic may inform teledermatology photography practice guidelines [20,21].

There is an inherent challenge in obtaining high-quality skin photographs through patients’ own webcams or phones. Many factors can influence teledermatology skin photo quality, including lighting, resolution, and camera quality. Although studies indicate patient acceptance of medical photography for teledermatology, these additional factors may impact the quality of photographs, which can negatively affect overall care and disease outcomes, and thus warrant further research.

### Future Directions

A recent US-based study indicated that verbal consent is now slightly preferred over written consent for medical photography—although importantly, a notable limitation of these results is the homogeneous study population (99% of participants identified as White) [11]. Further research into the possibly changing patient consent preference (written to verbal) among patient populations of all ethnicities is needed. Efforts to improve patient trust in nonphysician photographers, opposite-gender photographers, and EMR mobile applications will support clinic efficiency. Additional research regarding current perceptions of medical photography for various ethnic subgroups and on alternative interventions to improve patient acceptance of medical photography for Black and Latinx patients is also warranted. It may be worthwhile to investigate whether an informational booklet detailing the possible uses of medical photography and indicating the security of image storage improves Black or Latinx patient comfort with medical photography [4,10].

### Limitations

Limitations of this study include a relative lack of prior studies surrounding patient perceptions of medical photography in dermatology. Additionally, some of these studies are greater than 5 years old, and patient perceptions may have changed in recent years, especially in light of the COVID-19 pandemic and increased rates of teledermatology. Lastly, the use of the term “positive perceptions” as a blanket category was a limitation as the included studies did not have the exact same variables studied; however, creating a general category of “positive perceptions” helped to understand the larger picture of patient perceptions of medical photography in dermatology.

### Conclusions

The majority of published studies surveyed reported positive patient attitudes toward medical photography in dermatology. Patients felt that medical photography could improve their care and that research and teaching purposes were acceptable. Written consent forms listing all photo uses were preferred overall, with one recent 2020 US study [11] indicating a slight preference for verbal consent. Although physician and same-gender photographers were preferred, it is important to build patient trust in nonphysician and opposite-gender photographers to improve clinic efficiency [4]. Clinic-owned cameras with departmental record storage were preferred, but increased patient education regarding the safety of EMR phone applications is warranted. Disparities among ethnic groups were undeniable and were related to patient comfort with dermatologic medical photography. These disparities must be addressed to achieve equitable health outcomes for patients of all backgrounds. Future studies should be designed to capture the experiences of a wide array of ethnic subgroups to ensure health equity.

## Appendix

Multimedia Appendix 1 Table S1. Patient preferences of medical photography by category.

## Abbreviations

EMR: electronic medical record
SOC: skin of color
