# Supplementary material for: Patient Perceptions of Dermatologic Photography: Scoping Review
Source: JMIR Dermatol. 2022 Jan 26;5(1):e33361. doi: 10.2196/33361 (PMC10334897; doi:10.2196/33361)
Supplement: Multimedia Appendix 1 [file derma_v5i1e33361_app1.docx]

Table S1. Patient preferences of medical photography by category.

| **First Author** | **Informed Consent** | **Photographer Role and Badge** | **Gender** | **Photograph Capture Method** | **Image Storage** | **Image Uses & Identifiers** | **Mental Wellbeing and Trust** | **Ethnic Findings (FROM LEGER STUDY)** |
| --- | --- | --- | --- | --- | --- | --- | --- | --- |
| Hacard F. | Written Consent  Adult-80.2%  Peds-89.4%  Oral Consent  Adult-87.9%  Peds-64.5% | Adult survey  Professor-98.6% Physician-96.5% Resident-98.7%  Med Student-93.9%  Nurse-88.5%  Identification badge-82% | No gender preference  Adult survey- 92.3% Peds survey- 90.4% | Clinic/department only camera Adult survey- 93.8% Pediatric survey- 97.4% | Department records Adult-93.8%  Peds-97.8% Clinician personal computer  Adult-51.3% Peds-32.3% | Teaching  Adult-98.5%  Peds-91.9%  Research (deidentified) Scientific publications  Adult-67.4%  Peds-46.5% preferred over health websites (32.0%) | *Negative perception questions* Discomfort  Adult-11.4% Peds-3.3% "Dare not refuse"  Adult-7.5%  Peds-2.8% | Photos will not improve care  Latinx-19.7%  Black-12.5%  Asian-6.9%  White-5.3%  Identifiable intimate body area for medical care  White-91.2%  Black-80%  Latinx-68.3%  Asian-60.6%  Clinic-owned camera  White-95.36%  Black-90%  Asian-90.6%  Latinx-77.3%  Teaching (deidentified or identifiable)  *White and Black patients more comfortable than Latinx or Asian patients  *White patients most comfortable with any photographer gender and identity (physician, medical student, professional photographer, and nurse/PA)  *Black and Latinx patients more uncomfortable with medical pictures |
| Leger MC. | Written Consent-78.4%  Oral Consent-14.1% | Physician-94.4% Nurse/Physician Assistant (PA)-43% Medical Student-79.6% | Preferences  Same gender-90.3% Opposite gender-73.5% | *Clinic or hospital-owned camera preferred *Cell phones least preferred | N/A | De-identified photos  Teaching-87.4% Textbook/journal-83.2% Medical consult-91% *Deidentified photos more important for comfort than image purpose | N/A |  |
| Hsieh C. | Written consent-58.7%  Oral consent-38.6% | N/A | N/A | Hospital camera-97.7% Personal camera-27.5% Smartphone-27.2% *Smartphones acceptable for teaching reference  *Professionalism-31.5% | Confidentiality-65.6% Photography security-56.8% Default uploading to cloud-33.8% | Diagnosis and treatment  Teaching-75% comfortable except when a sensitive body area is photographed) Research | N/A |  |
| Secker L.J. | N/A | N/A | N/A | Total body photography (TBP) | N/A | Melanoma detection (31.1%)*  *Significantly associated with self-skin exam instructions from nurse/doctor & if TBP led to physician decision about mole excision | Ashamed of photos (27.9%) |  |
| Soriano L.F. | N/A | Hospital staff (satisfied) | N/A | Patient phone (satisfied)  Doctor phone (neutral)  *Afro-Caribbean patients less satisfied with physician smartphone/hospital system | Hospital computer system (satisfied) | Smartphone email/message between physicians (satisfied)  Another doctor views images on hospital computer (satisfied)  Patient wants to view skin photos before showing another doctor (<50%) | Older patients more dissatisfied with smartphone photo transfer between physicians or hospital system |  |

| **First Author** | **Informed Consent** | **Photographer Role and Badge** | **Gender** | **Photograph Capture Method** | **Image Storage** | **Image Uses & Identifiers** | **Mental Wellbeing and Trust** | **Additional Findings** |
| --- | --- | --- | --- | --- | --- | --- | --- | --- |
| Wang Y. | Written consent-47.0%  Oral consent-46.4%  *List all photo uses | Attending Physician-81% Other Physician-25% Nurse-24% Medical Student-19% Identification badge-84% | No gender preference-67% | Clinic camera-63.3% Cell phones-0.6% Personal camera-16.0% No preference-20% | Department records-81% | Diagnosis and treatment-79% Teaching-67.0% Case discussion-92.0% | Medical photography discomfort-5% Want to see photographs-78.7% | N/A |
| Pasquali P. | Consent varied based on body region lesions Malignant-88.8% Extensive-85.1% Facial-78.4% Genital- 70.2% | Attending Physician-95.5% Other Physician-64.9% Nurse-66.4% Photographer-21.6% | N/A | N/A | N/A | Diagnosis in self-86.6% Diagnosis in others-74.6% Treatment monitor-88.1% Doctor consultations-86.6% *Discomfort over sensitive body areas such as genital region | Patients with doubt over photo use had no prior medical photography exposure-70% | N/A |
| Accetta J.L. | N/A | N/A | N/A | Smartphone acceptance after information sheet detailing secure storage-79% | Info sheet (secure storage)-higher photo acceptance | N/A | Privacy/confidentiality concerns | More accepting of physician smartphone in academic vs. private practice |
| Wyatt K.D. | Verbal consent-52%  Written consent-27%  No consent needed-21% | Provider vs professional photographer-75% neutral | N/A | Mobile device vs professional camera-58% neutral | Requested photo copies and patient education handout (app security and privacy) | Document finding-79%  Specialist advice-16%  One-on-one learning-90%  Class education-80%  National meeting-73%  Med journal/textbook-68%  Social media education-42% | Privacy/confidentiality concerns-3%  Comfort varied with sensitive body areas | N/A |
| Amirian A. | Informed consent (subtheme) | Physician directly involved in patient’s care–99.5%  All physicians–49.5%  Medical students–39%  Paramedical students–25% | Male patients gave more consent in using skin photos | Hospital camera–62.3%  Professional camera–22.8%  Personal phone camera–14.9% | N/A | Educational purposes (subtheme) | Major themes  Physician trust  Confidentiality  Sub-themes  Professionalism Covering patient faces | N/A |
| Pathoulas J.T. | N/A | N/A | N/A | Standardized scalp photography | N/A | Self-perceived alopecia severity  Degree of anxiety associated with hair loss  Motivation to continue treatment-98.3% | Decreased alopecia-associated anxiety-81.5% | N/A |
